# Is There Evidence for Neurocognitive Dysfunctions in Patients with Postnatal HIV Infection? A Review on the Cohort of Hemophilia Patients

**DOI:** 10.3389/fnhum.2014.00470

**Published:** 2014-06-24

**Authors:** Silvia Riva, Ilaria Cutica, Gabriella Pravettoni

**Affiliations:** ^1^Department of Health Sciences, University of Milan, Milan, Italy; ^2^Istituto Europeo di Oncologia (IEO), Milan, Italy

**Keywords:** hemophilia, neurocognitive dysfunctions, cognitive test, HIV, postnatally infection, assessment

## Abstract

The debate regarding neurocognitive functions in the early stages of HIV infection is still ongoing; different studies have reached contrasting conclusions, probably because many of them take into account different cohorts of patients. A main distinction is between HIV seropositive patients infected perinatally, and those infected postnatally. The aim of this paper is to review results on neurocognitive dysfunctions and other types of neurological involvement in a specific cohort of HIV+ patients infected postnatally: hemophilia patients. Such a review is relevant, as HIV seropositive patients infected postnatally are understudied with respect to patients infected perinatally, and as the results of the few studies aiming at comparing them are contrasting. Taken together, the 11 studies reviewed suggest the presence of both long-term neurocognitive dysfunctions and neurological alterations, such as the presence of atrophic changes and lesions in the white matter. The current review may offer new research insights into the neurocognitive dysfunctions in HIV-patients, and on the nature of such dysfunctions.

## Introduction

HIV infection is associated with a variety of neurocognitive dysfunctions, which may occur at each stage of infection, and which progress during the course of the illness, although such progression may vary depending on therapies. Before the introduction of highly active antiretroviral therapy (HAART), neurocognitive dysfunctions represented a frequent outcome in about 60% of patients (Reger et al., 2002; Ettenhofer et al., 2009). Since HAART became current best practice, the quality of life of patients has improved, and neurocognitive complications have been reduced (Reger et al., 2002; Antinori et al., 2007). Notwithstanding, several studies have highlighted difficulties in neurocognitive performance at different levels (York et al., 2001; Sacktor et al., 2002; Carey et al., 2006; Iudicello et al., 2008; Woods et al., 2008; Cattie et al., 2012; Giesbrecht et al., 2014) in HIV seropositive (henceforth, HIV+) patients; some authors have argued that such difficulties might be more related to the presence of important covariates, such as drug abuse, cranial traumas, and several psychological alterations rather than to the direct action of HIV virus. The longitudinal observation of these patients has given contrasting results: some studies have not shown any decline in neurocognitive functions (Selnes et al., 1990, 1992; Grassi et al., 1995) while others have (Ayuso-Mateos et al., 2000; Woods et al., 2008; Ettenhofer et al., 2009; Applebaum et al., 2010).

Although findings across the HIV literature are not entirely consistent, a number of studies show that HIV-infected patients present deficits in speed of information processing (Carey et al., 2006; Giesbrecht et al., 2014), in fine motor speed (Sacktor et al., 2002) in learning and memory (Carey et al., 2006; Maki et al., 2009), in attention (Giesbrecht et al., 2014), and in multiple domains of executive functioning such as cognitive flexibility, decision-making, and planning (Iudicello et al., 2008; Cattie et al., 2012).

The issue of neurocognitive dysfunctions in HIV+ patients has both a speculative and a social relevance. It is quite known that in the US, for example, HIV+ patients have been victims of discrimination at work and/or at school because they have been considered as disabled people with cognitive deficits (Harrison and McArthur, 1995).

In the last 10–15 years, a growing interest has developed in the investigation of neurocognitive dysfunctions in specific HIV+ populations, such as homosexuals, drug-addicted people, and hemophilic patients. The case of hemophilia is particularly interesting even though the available studies are few and explorative. Hemophilia is a genetically transmitted bleeding disorder that affects mostly males, and requires infusions of clotting factors derived from donors’ blood. Hemophilia may determine severe bleeding episodes throughout life (Gringeri et al., 2011; Lessinger et al., 2013) that cause progressive arthropathy and joint damage. The major impact is on mobility, which may impair working and social life (Riva et al., 2010). Particularly dangerous is the intracranial bleeding, which can cause several neurocognitive dysfunctions especially regarding coordination, gait, and motor function (Mitchell et al., 1997; Basu et al., 2010). Hemophiliacs treated with factor infusions before 1985 have been at risk of acquiring HIV (Brookmeyer and Goedert, 1989). Several studies have shown that about 60–80% of patients with hemophilia, exposed to infected blood concentrates, contracted the HIV virus (Goedert, 1995). As a consequence, neurocognitive dysfunctions may also occur in HIV+ hemophilia patients.

The interest in neurocognitive dysfunctions in hemophilia HIV+ patients is quite innovative in the literature. Several studies have been conducted on the neurodevelopmental effects of HIV on children and adolescents who were infected perinatally, but much less is known on individuals infected postnatally, as is the case with hemophilia patients. This cohort of patients is different for several reasons: children infected postnatally through transfusion, have a more protracted course of disease than those who were infected perinatally (Cohen et al., 1991; Loveland et al., 1994, 2000); furthermore, they usually lack several psycho-social risk factors, commonly associated with perinatal infection that may act as confounding variables. Some such factors are, for instance, the severe illness or death of a parent due to AIDS, or a lower socioeconomic status. Finally, the developmental course of postnatally infected individuals may differ from that of those who are perinatally infected, because of differences in rate of brain growth: for instance, Rubin et al. (1999) found different patterns of neurobehavioral development depending on age of infection, and Brouwers et al. (1995) found differences in structural brain abnormalities between perinatally and transfusion-infected children.

For all these reasons, the HIV+ hemophilia patient population offers a unique opportunity to study the effects of postnatally acquired HIV infection.

However, up to now, only a few studies have been conducted on the neurocognitive dysfunctions of hemophilia HIV+ patients, with conflicting results. Furthermore, most of these studies took into account the influence of psycho-social issues such as health-related quality of life (HrQoL) (e.g., Fischer et al., 2003), treatment satisfaction (e.g., Gringeri et al., 2006; von Mackensen et al., 2013) and caregivers’ burden (e.g., Dekoven et al., 2013), but did not take into account the presence of HIV infection.

The present review examines works conducted in hemophilia patients HIV+ by systematically reviewing the literature to describe neurocognitive dysfunctions and to describe similarities and differences in comparison with the findings identified by the mainstream medical literature on HIV.

We aim to: (i) review the neuropsychological studies in HIV+ hemophilia patients performed so far; (ii) summarize the opportunities and challenges for future studies in this context.

In the following sections, we briefly review the main findings in this area. Studies were identified by searching the electronic databases Ovid Medline (1979-present) and PubMed (1979-present) and scanning reference lists of articles. Limits included the English language and humans. The last search was run on March 1, 2014, using the following search terms: HIV and hemophilia in combination with intellectual disabilities, behavioral disorders, intelligence quotient (IQ), cognition, neuropsychological disorders, and cognitive impairments.

## Results

The details of the major studies covered below are summarized in Table 1.

**Table 1:** **Listing of existing neuropsychological studies on hemophiliacs HIV+ patients**.

Reference	Country; type of participants	Cognitive functions examined	Major neurocognitive findings
Blanchette et al. (2002)	Canada; cross-sectional study in children: 14 HIV+ and 11 HIV−hemophiliacs	IQ, language, visual memory, verbal memory, motor speed, spatial memory, fine motor skills	HIV+ hemophiliacs showed impairment in motor speed and fine motor skills in comparison with HIV-patients
Hilgartner et al. (1993)	USA; cross-sectional study in children and adolescents. Three hundred thirty-three patients aged 6–18: 207 HIV+ hemophiliacs and 126 HIV−hemophiliacs (from HGDS)	IQ, memory, language, attention, visual perception, spatial perception, fine motor skills, emotional functioning	Around the 50% HIV+ hemophiliacs showed scores 1 SD below expected levels in three functional areas in comparison with HIV−hemophiliacs: IQ, language, memory
Loveland et al. (1994)	USA; from HGDS: cross-sectional results	IQ, memory, language, attention, visual perception, spatial perception, fine motor skills, emotional functioning	HIV+ hemophiliacs obtained lower results in comparison with HIV−hemophiliacs in the following areas: non-verbal intelligence (IQ), spatial perception, memory (non-verbal), language, decreasing is a linear relationship with the decreasing of immune functioning
Loveland et al. (2000)	USA; from HGDS: longitudinal results	IQ, memory, language, attention, visual perception, spatial perception, fine motor skills	HIV+ hemophiliacs obtained lower results in comparison with HIV−hemophiliacs in the following areas: non-verbal intelligence (IQ), perception, memory (non-verbal), language, decreasing is a linear relationship with the decreasing of immune functioning
Nichols et al. (2000)	USA; from HGDS: longitudinal results	Adaptive behavior style (including communication skills), emotional functioning	Decline in communication skills. Decreasing is a linear relationship with the decreasing of immune functioning
Riedel et al. (1992)	Germany; cross-sectional study in adults. 181 HIV+, and 28 HIV−hemophiliacs	Visual memory, motor speed, language, attention	HIV+ hemophiliacs obtained lower results in comparison with HIV−hemophiliacs in the following areas: attention, visuoperceptual speed, visuomotor speed, memory (verbal), decreasing is a linear relationship with the decreasing of immune functioning
Sirois et al. (1998)	USA; cross-sectional study in children and adolescents aged from 7 to 19: 178 HIV+ and 120 HIV−hemophiliacs	IQ, language, memory, attention, visual memory, spatial perception, fine motor skills, coordination, adaptive behavior	No differences between groups (lower performances of HIV+ are associated with covariates such as academic problems, head trauma, parents’ level of education)
Thompson et al. (1996)	UK; cross-sectional study in adolescents: 31 HIV+, and 33 HIV−hemophiliacs plus 16 controls	IQ, motor skills, motor speed, language, memory	HIV+ hemophiliacs performed better on most tests than HIV−hemophiliacs
Turnbull et al. (1991)	South Africa; case-study with four adults HIV+ hemophiliacs	IQ, visual memory, spatial perception, visual perception	HIV+ hemophiliacs obtained lower results in comparison with HIV−hemophiliacs in higher mental functions
Watkins et al. (2000)	USA; from HGDS: longitudinal results	Attention	Sustained attention was significant below in HIV+ hemophiliacs in comparison with -patient independently from the levels of CD4+ cells
Whitt et al. (1993)	USA; cross-sectional study in children and adolescents: 25 HIV+ and 38 HIV−hemophiliacs	IQ, attention, motor skills, visual processing, language, memory	HIV+ hemophiliacs obtained lower results (1SD below) in comparison with HIV−hemophiliacs in the following areas: attention, visuoperceptual speed, visuomotor speed

Most of the 11 studies reviewed took into account children and/or adolescents, and 2 evaluated adult patients. Most of the studies are from the USA and Canada, with the exception of one conducted in South Africa and two in Europe (UK and Germany). The cognitive functions examined varied, and different types of neuropsychological tests were used. However, the most important abilities evaluated are attention, memory (verbal and spatial), and motor skill coordination.

### Neuropsychological findings

As shown in Table 1, findings are often inconsistent and difficult to summarize. The most atypical results are by Thompson et al. (1996); a possible explanation may be that the comparison among groups is affected by the different mean age of the three patient groups (patients in the HIV-group were significantly older than participants in the other two groups), and the neuropsychological tests were not age-scaled.

Several of the remaining studies and most of the early research findings on hemophilia and HIV are based on the Hemophilia Growth and Development Study (HGDS; Hilgartner et al., 1993). The HGDS was a multicenter study of the long-term effects of HIV infection on growth and neurodevelopment in HIV+ hemophiliac children, most of whom were asymptomatic for HIV disease at baseline, in comparison with a group of HIV− subjects with hemophilia of similar age. Cross-sectional data from the HGDS (Hilgartner et al., 1993; Loveland et al., 1994) and paralleled findings of contemporary studies (Whitt et al., 1993; Sirois et al., 1998) have shown that the largely asymptomatic cohort of HIV+ children and adolescents did not differ significantly from HIV− hemophilic controls on a variety of neuropsychological tests. However, as indicated in follow-up studies of HGDS patients by Loveland et al. (2000) and Nichols et al. (2000), there was a significant decline in neurocognitive functions over 5 years, directly related to a decline in immune functioning. Furthermore, Watkins et al. (2000), still within the framework of longitudinal results of HGDS, has indicated that HIV-infected children showed significant differences in sustained attention performance, as measured by the Continuous Performance Test (CPT).

Both from HGDS results and from other cross-sectional studies (Whitt et al., 1993; Sirois et al., 1998), different authors have concluded that the lower neuropsychological performance might be attributed to the hemophilia itself and not to HIV. According to these authors, children suffering from hemophilia have levels of general mental ability, which are comparable with those of the general population. However their decrease in specific cognitive functions might be linked to the burden of illness that causes arthropathy (motor speed coordination) or school absenteeism for medical treatment (language and memory). These results are in line with the recent review of Moser et al. (2013). Other studies, on the contrary, have attributed the presence of neurocognitive dysfunctions to the HIV+ condition. All such studies have divided HIV+ hemophiliacs into sub-groups according to patients’ immune functioning and the authors of such studies concluded that significant differences in several cognitive functions arise with the decrease of immunological functioning, and especially when the CD4+ cell counts ≤200. For example, Riedel et al. (1992) identified a linear association between the scarce neuropsychological function in attention, motor skills, visual performance, and the decrease of CD4+. Similarly, according to Blanchette et al. (2002), HIV+ hemophiliacs have shown impairments in motor speed and fine motor skills, in comparison with those shown by HIV− hemophiliacs. Similarly, Nichols et al. (2000) concluded that HIV+ hemophiliacs present deficits in communication skills, related to the immune functioning system.

### Medical neurology and neuroimaging findings

The literature reported in Table 1, also gave us some interesting findings regarding medical neurology and neuroimaging. Most studies found neurological and neuroimaging tools to be essential in clinical diagnosis, useful in providing insights into the pathogenesis of HIV infection of the central nervous system (CNS), and in understanding the possible correlations with neurocognitive functions. However, in the field of hemophilia, these results are limited and heterogeneous. In terms of neurological and neuroradiological aspects, Loveland et al. (2000) identified neurologic abnormalities in HIV+ patients, such as progressive encephalopathies related to the age of infection. Furthermore, Sirois et al. (1998) identified a greater presence of decreased muscle bulk (around 50%) in the HIV+ hemophiliacs. Regarding neuroimaging, Mitchell et al. (1997), Mitchell et al. (1993), and Sirois et al. (1998) highlighted both the atrophic changes, as well as white matter lesions in young hemophilia HIV+ patients detected by magnetic resonance imaging (MRI).

Outside the HGDS context, two works deserve mention (although not inserted in the table). Rahemtulla et al. (1986) evaluating the clinical spectrum of the CNS in adult HIV+ patients with hemophilia, described the presence of diffuse subacute encephalitis while Hoots et al. (1998) identified diffuse muscle atrophy on neurological examination.

## Discussion

Taken together, the studies reviewed suggest some evidence in common, although the issue is still explorative. Studies from different countries, including large cohorts as the HGDS, report a long-term decline in neurocognitive functions in around 15–50% of patients (Loveland et al., 1994; Sirois et al., 1998). Decline seems to be proportional with the decrease of immunological functioning, especially when the CD4+ cell counts ≤200 (Riedel et al., 1992; Nichols et al., 2000; Blanchette et al., 2002), even though other studies have concluded that the decline is related to hemophilia *per se* (Hilgartner et al., 1993; Whitt et al., 1993; Loveland et al., 1994; Sirois et al., 1998).

Discrepancies among HIV+ and HIV− groups were found mainly in attention, memory performance, and visuomotor processing. However, neuropsychological test batteries differ among studies, in part because there is no agreement on what can be considered an abnormal test result (Schmälzle et al., 2011; Risko et al., 2012).

Results show that the type of neurocognitive dysfunctions in hemophilia HIV+ patients do not significantly differ from other HIV cohorts with the exception of the executive functioning’s domain, which appear to be unaltered in this group of patients. However, the origin of such decline is not yet identified unanimously and the debate is still open.

Summarizing, we may conclude that neuropsychological assessment, neuroradiology examinations, and MRI of the brain are important accessible tools in this clinical setting. Neuropsychological examination has been more reliably used in revealing the presence and character of neurocognitive dysfunctions, while neurologic and neuroimaging studies have proved helpful in identifying CNS tissue damage as a greater presence of decreased muscle bulk and lesions in white matter, which may become important markers in future research in the field.

### Future implications

Given the current state of the art, more studies are warranted on the mechanism behind the neurocognitive dysfunctions of HIV+ hemophiliacs: is it HIV-driven, or chronic immune-activation due to other unknown conditions, or the hemophilia itself, that leads to such a decline? The majority of the most recent studies seems to confirm the first hypothesis, but several uncertainties still remain. The clinical course of the neurocognitive dysfunctions is unidentified. For instance, it might be the result of a process that has been going on for years, or it might be the *exitus* of a subacute deterioration in which viral control in the CNS is suddenly lost. As reported by Schouten et al. (2011), we still do not know whether such deterioration will strike each HIV-infected hemophiliac, or a subset of patients at risk. Such a review can give us insight into the literature and compare different results in different cohorts of patients, but at the same time, many aspects about the neurology system in these patients are an unknown. The few studies available have provided information about the localization, the character, and the magnitude of the disease present in neurocognitive dysfunctions but this information is still fragmented. Thus, so far we are largely unable to elucidate and interconnect all the aspects related to neurocognitive dysfunctions. However, the results of the present review can be associated with those results identified in the general population with HIV.

As the data collected are not homogeneous, it is necessary to enhance the discussion around HIV and hemophilia from both a cognitive and a psycho-social point of view. Indeed, studies are still lacking on the impact of living with two chronic diseases, where both the conditions have some similarities and represent an evident burden on the patient’s life. As is well documented in the literature, HIV and hemophilia have a very significant psycho-social impact on patients, their families, and society in general, including psychological, cognitive, emotional, and social effects. These illnesses have important and durable effects on the individual, especially in the transition from adolescence where patients need to address a number of adaptations, ranging from greater demands for self-management of their health care, the impact of their illness on their emerging sexuality and independence, and the often difficult transition from familiar pediatric health care settings to unfamiliar adult providers (e.g., Brown et al., 2000; Pumariega et al., 2006; Steele et al., 2007; Bullinger and von Mackensen, 2008; Riva et al., 2010; Schmälzle et al., 2011; Dekoven et al., 2013; von Mackensen et al., 2013).

Today, the management of hemophilia has improved and patients, at least in western countries, have at their disposal several types of coagulation factor treatment and plasma-derivate concentrates. However, the geography of hemophilia is not the same around the world and over 80% of the world’s hemophilia patients receive inadequate or no therapy (Evatt et al., 1999; Kessler, 2005; Young, 2012). In several countries, patients are still at risk of high mortality due to either hemophilia or HIV. Therefore, the present review also aims to highlight hemophiliac patients’ conditions at the center of society’s economic and ethical concerns, and of the policy agenda.

## Conflict of Interest Statement

The authors declare that the research was conducted in the absence of any commercial or financial relationships that could be construed as a potential conflict of interest.
